# Prospect of positron emission tomography for abdominal aortic aneurysm risk stratification

**DOI:** 10.1007/s12350-021-02616-8

**Published:** 2021-05-11

**Authors:** Richa Gandhi, Michael Bell, Marc Bailey, Charalampos Tsoumpas

**Affiliations:** 1 Leeds Institute of Cardiovascular and Metabolic Medicine, School of Medicine, University of Leeds, 8.49 Worsley Building, Clarendon Way, Leeds, LS2 9NL United Kingdom; 2 Present Address: Brain Health Imaging Centre, Centre for Addiction and Mental Health, 250 College Street, Toronto, Ontario M5T 1R8 Canada

**Keywords:** Positron emission tomography, radioisotopes, abdominal aortic aneurysm, prognosis

## Abstract

Abdominal aortic aneurysm (AAA) disease is characterized by an asymptomatic, permanent, focal dilatation of the abdominal aorta progressing towards rupture, which confers significant mortality. Patient management and surgical decisions rely on aortic diameter measurements via abdominal ultrasound surveillance. However, AAA rupture can occur at small diameters or may never occur at large diameters, implying that anatomical size is not necessarily a sufficient indicator. Molecular imaging may help identify high-risk patients through AAA evaluation independent of aneurysm size, and there is the question of the potential role of positron emission tomography (PET) and emerging role of novel radiotracers for AAA. Therefore, this review summarizes PET studies conducted in the last 10 years and discusses the usefulness of PET radiotracers for AAA risk stratification. The most frequently reported radiotracer was [^18^F]fluorodeoxyglucose, indicating inflammatory activity and reflecting the biomechanical properties of AAA. Emerging radiotracers include [^18^F]-labeled sodium fluoride, a calcification marker, [^64^Cu]DOTA-ECL1i, an indicator of chemokine receptor type 2 expression, and [^18^F]fluorothymidine, a marker of cell proliferation. For novel radiotracers, preliminary trials in patients are warranted before their widespread clinical implementation. AAA rupture risk is challenging to evaluate; therefore, clinicians may benefit from PET-based risk assessment to guide patient management and surgical decisions.

**Supplementary Information:**

The online version contains supplementary material available at 10.1007/s12350-021-02616-8.

## Background

Abdominal aortic aneurysm (AAA) disease is characterized by localized, irreversible dilatation of the abdominal aorta from a normal diameter of approximately 10–20 mm to an aneurysmal diameter of 30 mm or greater. AAA is asymptomatic, yet simultaneously progressive towards rupture and profound internal bleeding, resulting in a high mortality rate in the range of 59–83% for patients with ruptured AAA who cannot access a hospital promptly and do not undergo surgery.^1^–^3^ From 2005 to 2012, 39,740 aneurysm-associated deaths occurred in England alone.^4^ Various environmental and genetic risk factors contribute to the development and progression of AAA; the predominant risk factors of AAA include advanced age, male sex, smoking, and elevated blood pressure.^1^,^5^,^6^ Emerging evidence also suggests that diabetes may play a protective role in patients who are susceptible to developing AAA; however, this correlation requires further investigation.^7^,^8^

## Scope of the clinical problem

Early-stage AAA does not manifest with any apparent signs or symptoms; therefore, it is usually diagnosed incidentally during unrelated medical check-ups or through screening programs, which have been formally launched in several countries including the UK, the US, and Sweden.^9^–^12^ The National Health Service AAA Screening Programme was fully implemented in 2013 in England to offer routine ultrasound scanning (USS)-based screening for all men 65 years of age and older. This program was established based on the results of the UK Multicentre Aneurysm Screening Study, which demonstrated a nearly 50% reduction in AAA-related mortality risk after 13 years in men invited for screening compared with that in men who did not undergo screening (0.66% vs 1.12%). This reduction was also found to be associated with an incremental cost-effectiveness ratio of £7,600 per quality-adjusted life years gained at 10 years.^13^,^14^ With a maximum reported sensitivity of 95% and specificity of nearly 100%, USS is predominantly used in the clinic to screen for AAA because of its ease of operability, low costs, low post-imaging workload and instantaneous results, and widespread availability.^3^ It is important to note here that although there is extensive ongoing research to establish more reliable and informative risk markers of AAA, the only indicator that is used consistently in the clinic is aortic size, which is derived from serial USS, or occasionally, computed tomography (CT). This is the simplest and most convenient parameter to extract using USS, but may not always accurately reflect prognosis, as small AAA has been reported to rupture, while large AAA has been reported as remaining intact.^15^ Therefore, the inability to accurately predict the prognosis of patients with AAA and stratify patients according to AAA risk remains a significant unmet clinical challenge.

In this context, molecular imaging may have a significant role in assessing AAA evolution. Positron emission tomography (PET) imaging offers a means to quantitatively measure molecular mechanisms related to AAA even at early stages as an adjunctive tool to USS-based screening and surveillance. PET is an attractive functional modality to reveal the underlying molecular changes that occur before the more apparent anatomical changes associated with AAA progression. The use of PET with a suitable radiotracer may then successfully help personalize a surveillance regimen or define a more precise intervention threshold to prevent aortic complications. Furthermore, it may contribute to the discovery of an appropriate pharmacological treatment for AAA, as currently, the only viable treatment option available is surgical intervention, the indication for which is based solely on the aortic size, as measured using USS. Establishing a pharmacological treatment that could reduce or even reverse AAA progression is a strategic aim of AAA-related research, but this necessitates a stratification imaging biomarker that could (a) help select patients who would benefit from such treatment, (b) predict AAA growth and rupture and refine watchful waiting regimens, (c) determine patient-specific intervention thresholds, or (d) be applied in all the aforementioned applications. Therefore, this review summarizes preclinical and clinical PET studies published in the last 10 years and discusses the usefulness of PET for AAA risk stratification.

## Preclinical studies

Much of our pathological understanding of AAA in patients is based on aortic tissue acquired during surgery (i.e., late-stage disease); hence, theories derived from these samples may not accurately represent the mechanisms of early-stage disease, when a novel pharmacological therapy may be administered. Preclinical studies of AAA are the first step in achieving a more complete picture of early-stage AAA pathophysiology, which may help establish stratification biomarkers that may be translated to the clinic. Table 1 presents the details of all preclinical PET studies of AAA published between 2010 and 2020.

**Table 1:** Preclinical PET studies of AAA conducted between 2010 and 2020.

Refs.	Species	Radiotracer	Targeted biomarker	Key conclusions
65	Rats	[^18^F]FDG, [^11^C]PBR28	Aortic wall inflammation	AAA wall inflammation can be detected using [^18^F]FDG and [^11^C]PBR28
39	Rats	[^18^F]FDG	Rupture prediction	Increased pre-rupture glucose uptake may be associated with increased inflammation in the ruptured AAA wall
17	Rats	[^64^Cu]DOTA-ECL1i	Expression of chemokine receptor 2	There is no change in [^64^Cu]DOTA-ECL1i uptake from day 7 to day 14 in PPE AAA, regardless of aortic diameter, and there is approx. 2× more uptake in AAA that proceeds to rupture
19	Mice	[^18^F]FLT	Cell proliferation	[^18^F]FLT uptake is increased during the active growth phase of AngII AAA compared to that in saline control mice and late-stage AAA
52	Mice	[^18^F]FPPRGD2	α_v_β_3_ expression	[^18^F]FPPRGD2 uptake in AAA may correlate with vascular inflammation and neoangiogenesis but not with AAA diameter
25	Mice	[^18^F]CLIO	Macrophages	[^18^F]CLIO may be used to quantify macrophage content in AAA
66	Rats	[^18^F]FDG	Intraluminal thrombus occurrence	Increased [^18^F]FDG uptake and growth may be associated with intraluminal thrombus occurrence in AAA
41	Rabbits	[^18^F]FDG	AAA progression	Inflammation plays a key role early in AAA development
24	Mice	[^18^F]NaF	Vascular microcalcification	[^18^F]NaF uptake is increased in AngII AAA preceding significant aortic expansion on days 14 to 28, and hydroxyapatite application aggravates AngII AAA
67	Rats	[^18^F]FDG, [^18^F]FCH, [^18^F]DPA714	Correlation between aortic wall inflammation and histopathological analysis	[^18^F]FDG may have higher sensitivity than that of [^18^F]FCH and [^18^F]DPA714 in detecting activated leukocytes in the aneurysmal wall
18	Mice	[^64^Cu]NOTA-TRC105-Fab	Angiogenesis	[^64^Cu]NOTA-TRC105-Fab uptake indicates regions of increased angiogenesis in AAA, based on cluster of differentiation 105 expression

*AAA*, abdominal aortic aneurysm; AngII, angiotensin II; *[*^*64*^*Cu]DOTA-ECL1i*, [^64^Cu]-(1,4,7,10-tetraazacyclododecane-1,4,7,10-tetraacetic acid)-extracellular loop 1 inverso; [^*18*^*F]FCH*, [^18^F]fluoromethylcholine; [^18^F]CLIO, [^18^F]-cross-linked iron oxide; *[*^*18*^*F]FDG*, [^18^F]fluorodeoxyglucose; [^*18*^*F]FLT*, [^18^F]fluorothymidine; *[*^*18*^*F]NaF*, [^18^F]-sodium fluoride; *PET*, positron emission tomography

Preclinical studies are conducted with the aim of better matching animal models to the equivalent stage of human AAA. Calcification, proliferation, angiogenesis, and inflammation are some key processes that contribute to the AAA pathogenesis^16^; elucidating how these mechanisms are inter-connected at various stages of AAA progression can guide the development of more longitudinally representative preclinical models. For example, English et al. demonstrated that the uptake of [^64^Cu]DOTA-ECL1i shows little variation between days 7 and 14 post-aneurysm induction in the porcine pancreatic elastase-induced AAA murine model, reflecting non-fluctuating levels of vascular wall inflammation during this period.^17^ Another [^64^Cu]-based radiotracer, [^64^Cu]NOTA-TRC105-Fab, has been shown to exhibit high uptake in the calcium-induced murine model, indicating increased angiogenic activity; however, its high residual blood pool activity even 24 hours post-injection remains a key limitation.^18^ Using the angiotensin II-induced AAA murine model, Gandhi et al. showed that there is increased [^18^F]fluorothymidine ([^18^F]FLT) uptake on day 14 post-aneurysm induction, but this uptake is significantly reduced on day 28.^19^ These findings have important implications in supporting the idea of early-stage cellular remodeling that may drive disease progression, a notion that has been introduced and deliberated by several groups.^20^–^22^ In the future, knowledge gained from these studies may facilitate early-stage risk stratification; however, further preclinical and clinical investigations are warranted to reinforce this theory.^23^ Li et al. demonstrated that the uptake of [^18^F]-labeled sodium fluoride ([^18^F]NaF), reflecting microcalcification, is increased between days 14 and 28 in the angiotensin II-induced AAA murine model preceding notable aortic expansion.^24^ In the same model, Nahrendorf et al. showed that the uptake of [^18^F]-cross-linked iron oxide ([^18^F]CLIO) in AAA is associated with the risk of AAA growth and rupture.^25^ However, a key drawback to the use of nanoparticle-based radiotracers such as [^18^F]CLIO is their extended circulation period, necessitating delayed imaging to avoid capturing the blood pool activity that may mask AAA uptake. Radiotracers consisting of smaller nanoparticles with a shorter clearance time may evade this issue. These are just a few examples of studies that have attempted to investigate the time course of biological events in AAA, which can help develop more informative preclinical models of AAA that are easily translatable to the clinic.

Nevertheless, the translation of preclinical PET research to the clinic is not free of hurdles. There is an overwhelming cost of introducing new radiotracers in the clinic, along with long-standing regulations that may prevent or delay the implementation of radiotracers not readily used for other applications such as cancer diagnostics. Few centres have easy access to a cyclotron for radiotracer production, thereby largely limiting centers to using radiotracers with sufficiently workable half-lives such as radiotracers labeled predominantly with [^18^F]; instead, alternatives that do not require a cyclotron for production such as [^68^Ga]-labeled radiotracers may be employed.^26^ Moreover, the translational success of preclinical findings may then be influenced by several factors, such as differences in toxicology and pharmacokinetics^27^; gaining a better understanding of these factors will ultimately benefit the clinical translation of important preclinically validated biomarkers.

## Clinical studies

Over the last several decades, the incidence of AAA rupture has decreased. This is largely attributed to the implementation of AAA screening programs and novel therapeutic interventions, such as endovascular aortic repair (EVAR). That being said, the low rupture rate of small aneurysms and risks associated with surgery do not sufficiently rationalize the routine repair of small AAA.^28^ Clinical guidelines on the use of EVAR have been amended often, owing to its reduced long-term durability and moderate long-term survival benefits.^29^ Therefore, by non-invasively detecting established biological targets, such as inflammation with [^18^F]fluorodeoxyglucose ([^18^F]FDG) PET, clinicians may be able to identify patients with a high risk of clinical events and those who may benefit the most from elective repair. Generally, PET radiotracers approved for non-AAA applications have facilitated clinical studies of AAA. Table 2 presents the details of all clinical PET studies of AAA conducted between 2010 and 2020, revealing as expected that the predominant radiotracer in use is [^18^F]FDG.

**Table 2:** Clinical PET studies of AAA conducted between 2010 and 2020.

Refs.	Radiotracer	Targeted biomarker	Key conclusions
44	[^18^F]FDG	Glucose metabolism	Metabolic activity levels may not correlate with aortic size or differ between aneurysms vs controls
42	[^18^F]FDG	Correlation between inflammation and histological analysis	[^18^F]FDG uptake in the aneurysmal wall may be associated with an active inflammatory process involving proliferating leukocytes and increased circulating C-reactive protein
68	[^18^F]FDG	Circulating microRNAs	Specific microRNAs are significantly correlated with [^18^F]FDG uptake in the aneurysmal wall and may be directly involved in AAA instability
47	[^18^F]FDG	Prediction of complications after EVAR	Aortic [^18^F]FDG uptake may be a predictor of post-EVAR complications
54	[^18^F]FCH	Incidental AAA detection in patients with prostate cancer	[^18^F]FCH PET/CT may be an effective approach for secondary prevention and stratification of AAA in patients with prostate cancer
50	[^18^F]NaF	AAA growth and clinical outcomes	[^18^F]NaF uptake may help identify advanced AAA and may be correlated with aneurysm growth and clinical AAA events that differ from established risk factors
40	[^18^F]FDG	Structural stress	Increased [^18^F]FDG is associated with high mechanical stress of thick intraluminal thrombus in AAA
55	[^18^F]FDG	Inflammation	There is greater inflammation (based on [^18^F]FDG PET) and calcification (based on the Agatston score) in patients with AAA than in patients with atherosclerosis
38	[^18^F]FDG	Future aneurysm expansion	AAA with lower metabolic activity may be more likely to expand
69	[^18^F]FDG	Correlation between CT texture analysis data and metabolism	CT textural data may reflect AAA metabolism measured by [^18^F]FDG PET
46	[^18^F]FDG	Inflammation	[^18^F]FDG PET/MRI may be used to assess inflammation in asymptomatic AAA, although the hotspots of [^18^F]FDG uptake and late gadolinium enhancement are not always aligned
70	[^18^F]FDG	Decision to perform intervention	[^18^F]FDG uptake may be related to the clinical conditions of patients with AAA who require intervention
71	[^18^F]FDG	Correlation between metabolic activity and non-linear finite element analysis	Greater [^18^F]FDG activity may be associated with increased mechanical stress in the AAA wall
72	[^18^F]FDG	Correlation between [^18^F]FDG uptake and asymptomatic non-inflammatory AAA	Low [^18^F]FDG uptake in asymptomatic AAA reflects the loss of tissue structure and reduced cell density
73	[^18^F]FDG	Vascular inflammation and correlation with USPIO MRI uptake	[^18^F]FDG uptake may indicate macrophage glycolytic activity in AAA but shows a modest correlation with USPIO uptake
64	[^18^F]FDG	AAA progression	[^18^F]FDG PET/CT and PET/MRI do not correlate with disease symptoms, AAA progression, or dissection
74	[^18^F]FDG	Relationship between inflammation and risk factors	Vascular inflammation plays a role at all stages of AAA, which may involve the local result of systemic inflammation
75	[^18^F]FDG	Metabolic changes	Metabolic changes in AAA may follow a cyclic pattern, similar to that observed with changes in maximal aortic diameter
76	[^18^F]FDG	Infection	[^18^F]FDG PET is useful to diagnose infected AAA
43	[^18^F]FDG	Biomechanical properties	Increased [^18^F]FDG uptake is correlated with AAA location, wall stress, and patient risk factors
77	[^18^F]FDG	Asymptomatic aneurysmal uptake	[^18^F]FDG uptake may be rare in patients with AAA of diameter approaching the surgical threshold
78	[^18^F]FDG	Correlation between glucose metabolism and partial volume correction	Partial volume correction may be necessary to quantitatively stratify patients for AAA repair
79	[^18^F]FDG	Inflammation	[^18^F]FDG PET cannot be used to detect chronic inflammation in asymptomatic aneurysms
80	[^11^C]PK11195, [^11^C]-d-deprenyl	Inflammation	Inflammation in AAA cannot be detected using [^11^C]PK11195 or [^11^C]-d-deprenyl
53	[^18^F]fluciclatide	α_v_β_3_ expression	α_v_β_3_ integrin expression in AAA may be visualized *ex vivo* using [^18^F]fluciclatide
45	[^18^F]FDG	Aortic wall inflammation	Active aortic wall inflammation may contribute to AAA progression and rupture
81	[^18^F]FDG	Wall stress and metabolic activity	High AAA wall stress and accelerated metabolism may be associated

*AAA*, abdominal aortic aneurysm; *CT*, computed tomography; EVAR, endovascular aortic repair; [^18^F]FCH, [^18^F]fluoromethylcholine; [^*18*^*F]FDG*, [^18^F]fluorodeoxyglucose; *[*^*18*^*F]NaF*, [^18^F]-sodium fluoride; MRI, magnetic resonance imaging; *PET*, positron emission tomography; *USPIO*, ultrasmall superparamagnetic iron oxide

## Controversy of [^18^F]FDG

[^18^F]FDG is the most common radiotracer utilized for PET imaging. As presented in Table 1 and Table 2, there has been an extensive focus on markers of inflammatory activity, evidenced by the overwhelming predominance of [^18^F]FDG PET studies: 5 of 11 preclinical and 24 of 28 clinical studies. With its widespread availability and well-established safety profile, [^18^F]FDG is a typically universal starting point for clinical PET research. The intracellular distribution of [^18^F]FDG is associated with the degree of metabolic activity and inflammation in regions of uptake.^30^ [^18^F]FDG uptake on PET has been demonstrated to correlate with macrophage density in plaques, cardiovascular risk factors, the Framingham risk score, and various inflammatory and glycolysis-related biomarkers, such as glucose transporters 1 and 3 and total lesion glycolysis.^31^–^33^ Yet, a patient receiving anti-inflammatory drugs has been reported to develop AAA that rapidly expanded and ruptured^34^; this case suggests that inflammation may be fallible as a single indicator of patient prognosis and may need to be supplemented with other biomarkers. A downside to the advantage of [^18^F]FDG highlighting all regions of active glucose metabolism is that, as a result, it is difficult to differentiate disease-specific activity. Moreover, the uptake of [^18^F]FDG can be influenced by conditions of disease microenvironments, such as hypoxia or increased myocardial muscle activity, or the efficiency with which the microcirculation distributes the radiotracer.^35^–^37^

Both preclinical and clinical studies of gold-standard [^18^F]FDG PET to assess glucose metabolism and inflammation in AAA have yielded varying results of both increased and variable [^18^F]FDG uptake.^38^–^41^ Some studies have shown that [^18^F]FDG PET may be useful to predict AAA expansion and/or progression,^42^,^43^ while other studies have contradicted this.^38^,^44^ For example, Tsuruda et al. showed that increased aortic uptake of [^18^F]FDG in patients is associated with active aortic wall inflammation, which may contribute to AAA progression and rupture, while patients showing [^18^F]FDG uptake in AAA revealed no correlation with aortic expansion 12 months later in a study by Kotze et al.^38^,^45^ Moreover, Nchimi et al. and Huang et al. demonstrated that [^18^F]FDG uptake correlates positively with wall stress and strength in AAA, whereas Barwick et al. found no significant difference in aortic uptake of [^18^F]FDG between patients with infra-renal AAA and patients without AAA.^40^,^43^,^44^

In a study of [^18^F]FDG PET and contrast-enhanced magnetic resonance imaging (MRI), Kuzniar et al. demonstrated that the hotspots of [^18^F]FDG uptake and late gadolinium enhancement rarely coincide in AAA, although both are associated with aneurysm growth, raising interesting questions about the distribution of cellular activity in AAA.^46^ Collectively, these findings contribute to a complicated story of glucose metabolism in AAA. This scenario is further confounded by the lack of consistency in the type of AAA presentation studied, such as asymptomatic, symptomatic, end-stage, and/or early-stage AAA. Meanwhile, [^18^F]FDG PET may have a promising role in identifying patients who may be at risk of experiencing endoleaks after undergoing EVAR.^47^–^49^ Validation of these prior studies would benefit from larger prospective studies investigating patients with a consistent type of AAA presentation. Further data corresponding to kinetic modeling and corrections for blood flow and volume would also be informative, lending to more comprehensive analysis and validation. Therefore, supplementing the information yielded by [^18^F]FDG with information from other radiotracers may facilitate a more detailed understanding of the molecular workings of AAA.

## Emerging radiotracers

AAA comprises an intricate environment of various biological pathways; therefore, other PET radiotracers are also promising in the context of AAA. The sodium fluoride imaging of AAA (SoFIA^3^) trial has shown that uptake of [^18^F]NaF, which reflects regions of calcification, in patients with end-stage asymptomatic AAA predicts AAA progression and rupture, providing proof-of-concept data for the feasibility of a non-[^18^F]FDG PET radiotracer for AAA stratification. The findings of the trial suggest that [^18^F]NaF PET/CT is an effective tool to identify AAA disease activity in patients, based on the localization of the radiotracer in regions of microcalcification, which in turn, is a susceptibility marker of aneurysm expansion and rupture early after AAA formation.^50^

A key point arising from this study is that aneurysm size is not necessarily a predictor of rupture; AAA growth may be non-linear and influenced by biomechanical processes that may not necessarily exhibit an obvious detectable pattern. The SoFIA^3^ trial demonstrated that [^18^F]NaF uptake is a positive predictor of aneurysm growth and clinical outcomes, which are independent but supplementary to classic clinical parameters such as aneurysm diameter.^50^ The findings of this trial must be considered in light of the confounding issue of spill-in contamination from the nearby bone into the aneurysm; thus, background correction techniques are likely necessary to provide more robust quantitative assessments of AAA using [^18^F]NaF.^51^ Alternative radiotracers that may prove to be useful include markers of other characteristics of AAA development, such as angiogenesis, using integrin- or endoglin-targeted agents.^18^,^52^,^53^ Markers of cell proliferation and chemokine receptors such as [^18^F]FLT and [^64^Cu]DOTA-ECL1i are promising, and preliminary trials in patients are warranted to determine if these approaches may translate to the clinic.^17^,^19^,^23^ [^18^F]fluoromethylcholine, which is commonly implemented in prostate cancer staging, may also be beneficial to incidentally detect AAA in patients with prostate cancer.^54^ With these radiotracer applications, PET remains an attractive modality to advance our understanding of AAA pathophysiology, while novel molecular tracer agents are introduced, and hybrid multimodality systems are improved. As PET is readily accessible and already being used in the clinic, continued research using PET has practical applications that are feasible for clinical translation.

## Outlook of PET for AAA assessment

AAA remains a significant cause of mortality due to aortic rupture. USS reveals anatomical changes in AAA size and shape; however, there remain gaps in our knowledge of the molecular changes that precede physical manifestation of AAA and in our ability to visualize these pathological mechanisms at the molecular level. Despite the large number of AAA studies conducted, our knowledge of AAA pathobiology is incomplete, and there remains a lack of concrete data to translate preclinical findings to clinical practice. The contributions of inflammatory activity, biomechanical disturbances, calcification, angiogenesis, and cell proliferation to aortic growth and aneurysm progression remain only partially understood; these may be clarified with the application of PET imaging utilizing specific biologically targeted radiotracers. A better understanding of these functional changes in early-stage AAA may help stratify patients based on rupture risk early in the disease course and guide appropriate treatment selection. In this context, the multifactorial pathophysiology of AAA in humans represents a growing area in cardiovascular research, largely because clinical studies of AAA are predominantly limited to using late-stage aortic tissue obtained during surgery, owing to the asymptomatic presentation of early-stage AAA and lack of intervention (and easy access to tissue) at this stage. Although several risk factors and potential contributors to the disease process have been identified, such as sex, age, comorbidities, and smoking, these are not yet sufficiently predictive when applied to individual patients as reliable and accurate assessment methods for these factors are not yet readily available. Thus, as management decisions are made based solely on aortic size, many patients are needlessly subjected to the risks of aortic repair, even in cases wherein an aneurysm might not have ruptured if left untreated.

As we move forward, a shift away from the size of an aneurysm as the sole indicator of AAA repair is perhaps required. The feasibility of alternative markers of AAA progression that may be more reliable must be investigated as adjuncts to traditional USS-based size information, including circulating biomarkers, four-dimensional or dynamic contrast-enhanced MRI findings such as vascular flow patterns, CT-based calcium scores, and haemodynamic parameters.^55^–^60^ Although anatomical imaging is mainly used in both preclinical and clinical imaging of AAA, molecular imaging is gaining increasing importance, and several new studies are anticipated in forthcoming years. Anatomical and molecular imaging techniques are not necessarily competitive; rather, they can be complementary in their usefulness for AAA management. Thus, PET offers a non-invasive means to detect and evaluate components of potentially contributing pathways in patients to better understand AAA development beyond morphological features. Ultimately, the most successful imaging tracer will reveal key aspects of AAA formation in humans, be used for patient risk stratification, and help assess treatment efficacy in novel clinical trials.^61^ Introducing a novel imaging tracer to the clinic is a lengthy and costly process, akin to drug research and development. Ultimately, novel radiotracers that may also be implemented in multiple diseases are more likely to undergo rapid and efficient clinical translation. Considering the rupture rate of small AAA, a large sample of patients would be required to demonstrate a novel agent’s value in assessing rupture risk for AAA smaller than 55 mm in diameter. Meanwhile, the presence of comorbidities such as heart and vascular diseases greatly affects a patient’s outcomes of surgical repair.^62^ Hence, a transitional step may be to first investigate patients with AAA larger than 55 mm in diameter, as they consequently carry a risk of complications, to identify who may benefit from repair in the presence of comorbidities.

## PET technology and methodology

The ability of PET to yield precise, longitudinal quantitative data makes it a strong contender for clinical translation in the context of AAA risk stratification. Improvements in spatial resolution and the application of partial volume correction and background activity adjustments may improve the accuracy and precision of PET analysis methods in the future.^63^ One setback of PET is the potential inter-analytical variability depending on how the aortic region of interest (ROI) is defined. Standardizing ROI definitions and analytical methods is imperative to overcoming this challenge, and a significant advantage of PET is that data can be reviewed and corrected post-acquisition for temporal and spatial resolution, something that USS that does not yet offer. The ability to perform kinetic modeling of PET data, which would elucidate radiotracer biodistribution in the blood circulation, further makes PET an attractive and useful technology for AAA assessment. The small size of arteries and vessel walls necessitates the coupling of PET with anatomical modalities to distinguish radiotracer uptake in the aorta from that in surrounding regions. Standalone PET is typically combined with CT to add a layer of anatomical information while compensating for the limited spatial resolution of PET. With their widespread availability and low associated costs, hybrid PET/CT systems continue to advance towards becoming a valuable prognostic and risk stratification tool; meanwhile, the combination of MRI and PET has also emerged, with the superior advantage of combining molecular imaging with substantial soft tissue contrast than that achieved using CT. With these new imaging systems, PET and MRI data are acquired simultaneously in a single scan, and the images are promptly co-registered, effectively reducing misalignment errors. More studies will confirm whether PET and MRI parameters are correlated or reveal different types of information.^46^,^64^ Nonetheless, technical developments in PET and hybrid PET systems will continually help our understanding of the pathophysiological factors contributing to AAA evolution.

## Conclusions

Despite substantial advances in AAA screening and management over the years, AAA remains clinically significant. Management of patients with AAA is currently based on morphological characteristics derived from USS-based surveillance, including aortic size and growth rate. USS provides reliable anatomical data in this regard. However, the classical anatomy-centered approach to AAA imaging is insufficient to comprehensively assess rupture risk and factors that may result in complications associated with repair. Emerging radiotracers with PET can provide complementary functional information at the molecular level. [^18^F]FDG PET studies have yielded equivocal results, urging the introduction of more relevant radiotracers. Importantly, the strength of PET lies in its ability to elucidate the molecular evolution of AAA, which can inform the need for surgical intervention or response to pharmacological therapy. Despite the low number of preclinical studies, these are important for pilot evaluations of novel agents and improved characterization of existing radiotracers prior to their clinical translation. Finally, for clinical translation, the standardization of analysis methods and improvements in hybrid technology are warranted. Therefore, in patients with AAA, the road ahead for PET imaging is promising to facilitate assessments of functional aortic characteristics that may help distinguish patients with rupture-prone AAA requiring surgery from patients with low-risk AAA for whom a watchful waiting approach may be sufficient.

## Supplementary Information

Below is the link to the electronic supplementary material.

Supplementary material 1 (PPTX 163 kb)

Supplementary material 2 (M4A 4914 kb)

## Abbreviations

AAA: Abdominal aortic aneurysm
CT: Computed tomography
EVAR: Endovascular aortic repair
[^18^F]FDG: [^18^F]fluorodeoxyglucose
[^18^F]FLT: [^18^F]fluorothymidine
[^18^F]NaF: [^18^F]-labeled sodium fluoride
MRI: Magnetic resonance imaging
PET: Positron emission tomography
SoFIA^3^: Sodium fluoride imaging of AAA
USS: Ultrasound scanning
