# Supplementary material for: Prospect of positron emission tomography for abdominal aortic aneurysm risk stratification
Source: J Nucl Cardiol. 2021 May 11;28(5):2272–82. doi: 10.1007/s12350-021-02616-8 (PMC8648657; doi:10.1007/s12350-021-02616-8)
Supplement: Supplementary file 1 — Supplementary material 1 (PPTX 163 kb) [file 12350_2021_2616_MOESM1_ESM.pptx]

## Slide 1
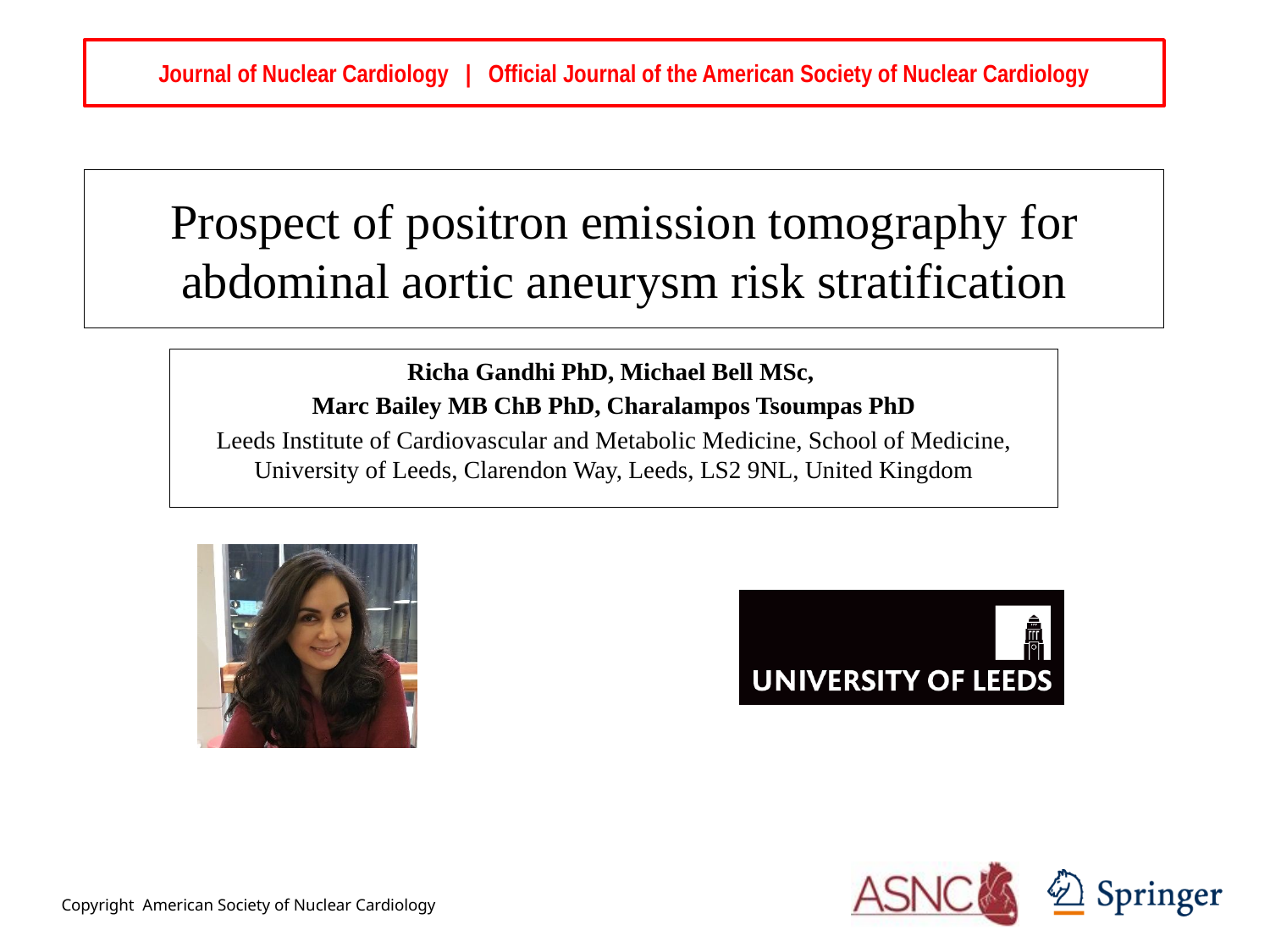

Journal of Nuclear Cardiology | Official Journal of the American Society of Nuclear Cardiology
# Prospect of positron emission tomography for abdominal aortic aneurysm risk stratification
Richa Gandhi PhD, Michael Bell MSc,
Marc Bailey MB ChB PhD, Charalampos Tsoumpas PhD
Leeds Institute of Cardiovascular and Metabolic Medicine, School of Medicine, University of Leeds, Clarendon Way, Leeds, LS2 9NL, United Kingdom
Copyright American Society of Nuclear Cardiology

## Slide 2
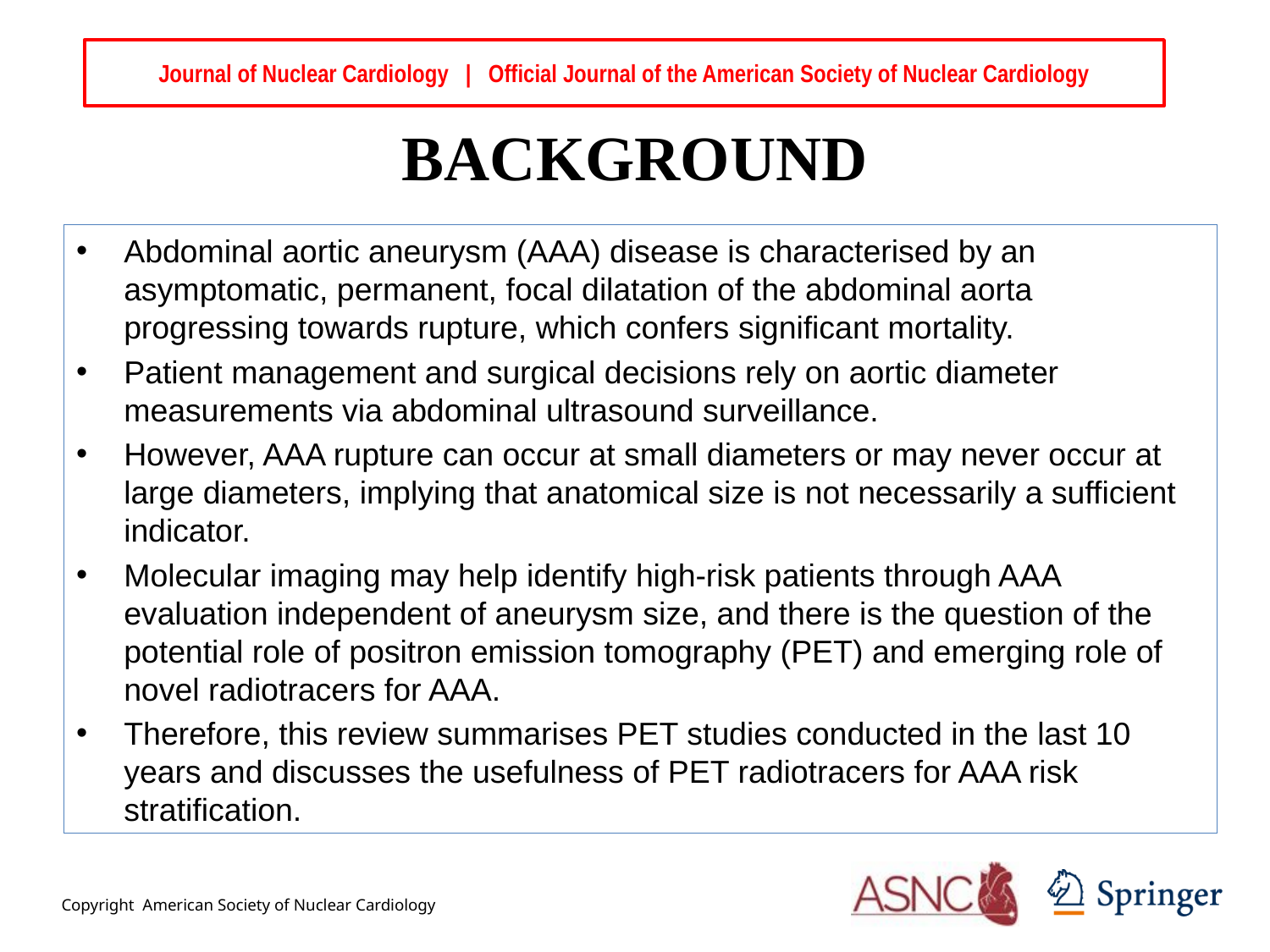

Journal of Nuclear Cardiology | Official Journal of the American Society of Nuclear Cardiology
# BACKGROUND
Abdominal aortic aneurysm (AAA) disease is characterised by an asymptomatic, permanent, focal dilatation of the abdominal aorta progressing towards rupture, which confers significant mortality.
Patient management and surgical decisions rely on aortic diameter measurements via abdominal ultrasound surveillance.
However, AAA rupture can occur at small diameters or may never occur at large diameters, implying that anatomical size is not necessarily a sufficient indicator.
Molecular imaging may help identify high-risk patients through AAA evaluation independent of aneurysm size, and there is the question of the potential role of positron emission tomography (PET) and emerging role of novel radiotracers for AAA.
Therefore, this review summarises PET studies conducted in the last 10 years and discusses the usefulness of PET radiotracers for AAA risk stratification.
Copyright American Society of Nuclear Cardiology

## Slide 3
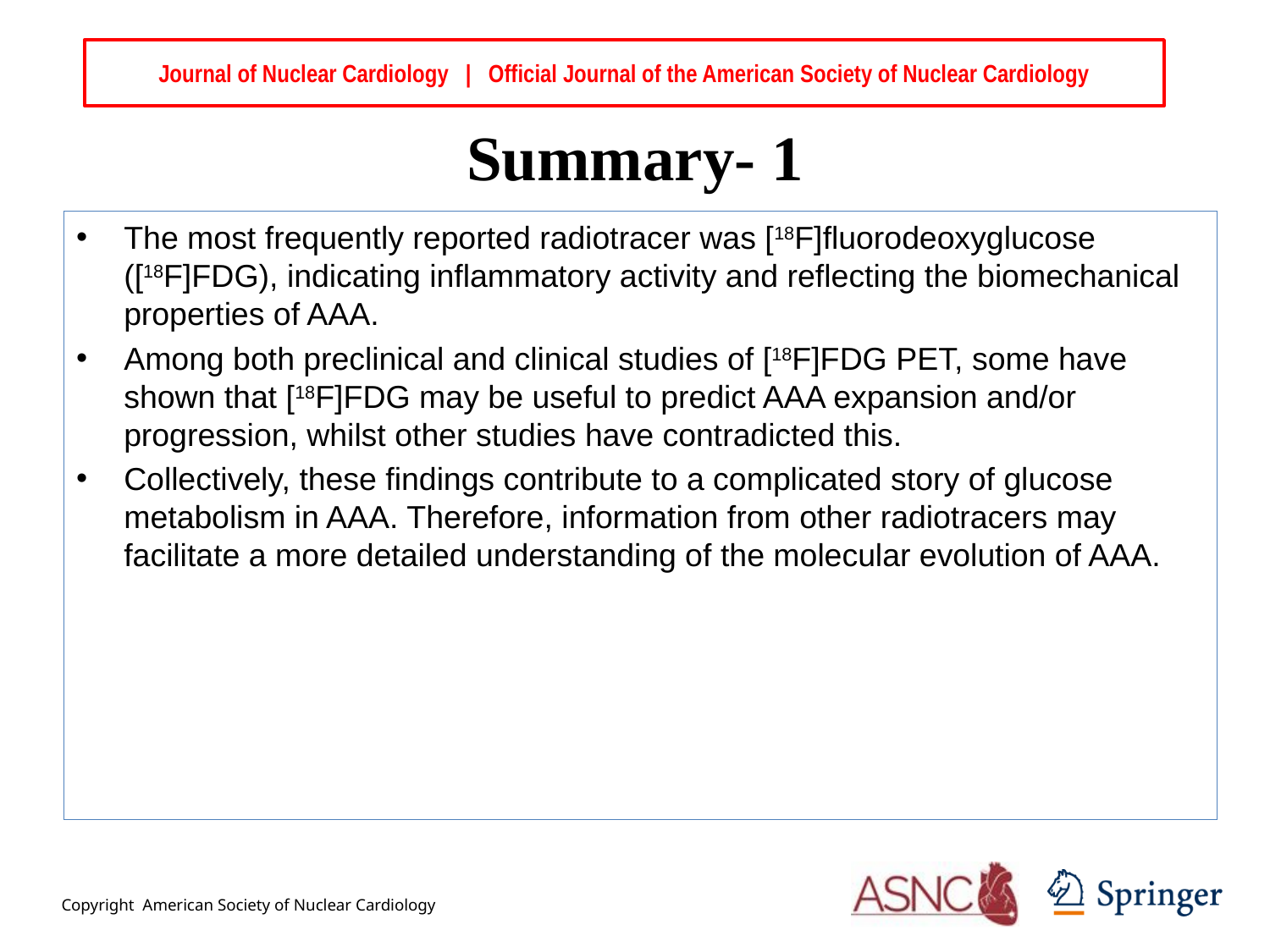

Journal of Nuclear Cardiology | Official Journal of the American Society of Nuclear Cardiology
# Summary- 1
The most frequently reported radiotracer was [18F]fluorodeoxyglucose ([18F]FDG), indicating inflammatory activity and reflecting the biomechanical properties of AAA.
Among both preclinical and clinical studies of [18F]FDG PET, some have shown that [18F]FDG may be useful to predict AAA expansion and/or progression, whilst other studies have contradicted this.
Collectively, these findings contribute to a complicated story of glucose metabolism in AAA. Therefore, information from other radiotracers may facilitate a more detailed understanding of the molecular evolution of AAA.
Copyright American Society of Nuclear Cardiology

## Slide 4
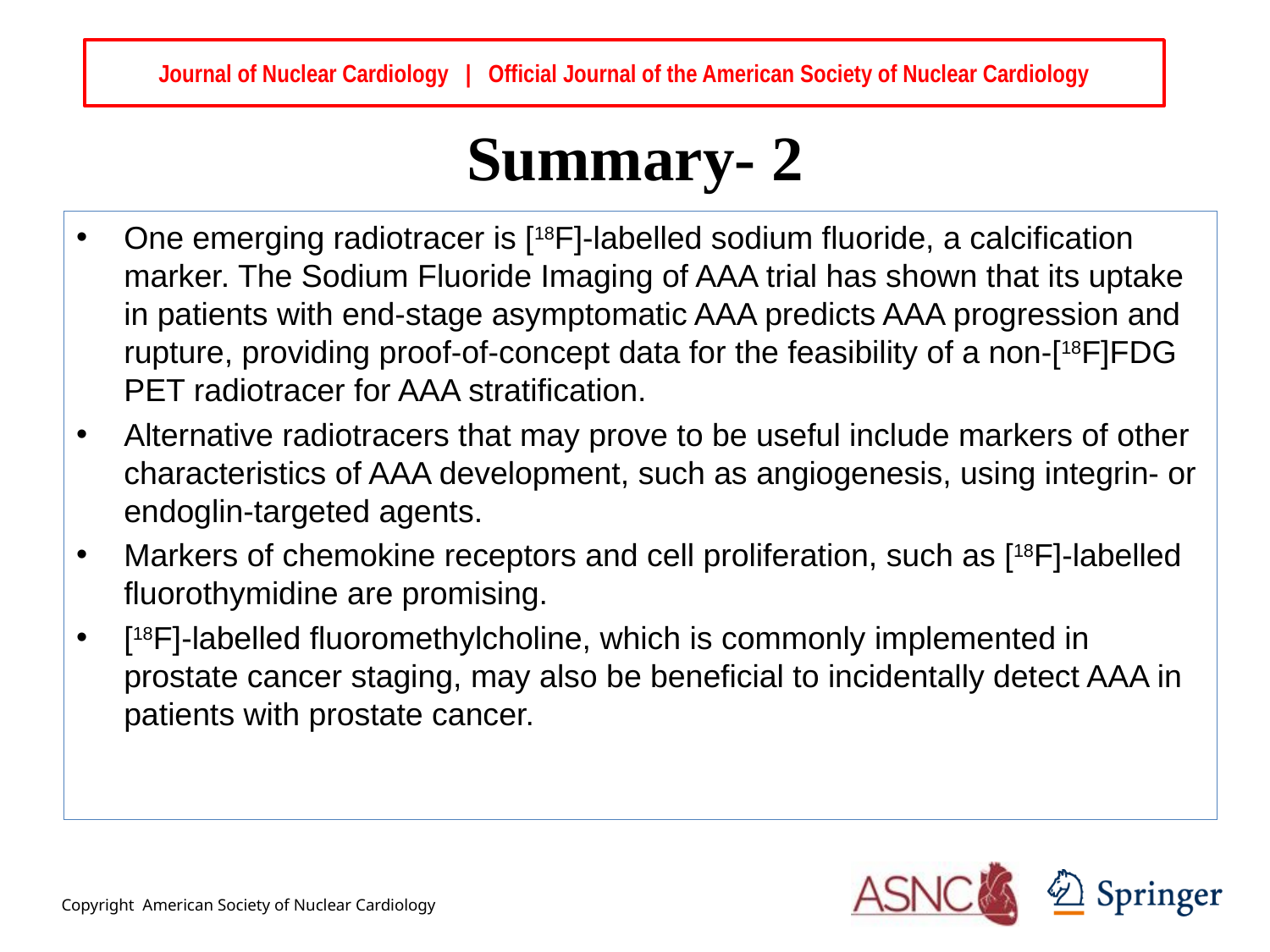

Journal of Nuclear Cardiology | Official Journal of the American Society of Nuclear Cardiology
# Summary- 2
One emerging radiotracer is [18F]-labelled sodium fluoride, a calcification marker. The Sodium Fluoride Imaging of AAA trial has shown that its uptake in patients with end-stage asymptomatic AAA predicts AAA progression and rupture, providing proof-of-concept data for the feasibility of a non-[18F]FDG PET radiotracer for AAA stratification.
Alternative radiotracers that may prove to be useful include markers of other characteristics of AAA development, such as angiogenesis, using integrin- or endoglin-targeted agents.
Markers of chemokine receptors and cell proliferation, such as [18F]-labelled fluorothymidine are promising.
[18F]-labelled fluoromethylcholine, which is commonly implemented in prostate cancer staging, may also be beneficial to incidentally detect AAA in patients with prostate cancer.
Copyright American Society of Nuclear Cardiology

## Slide 5
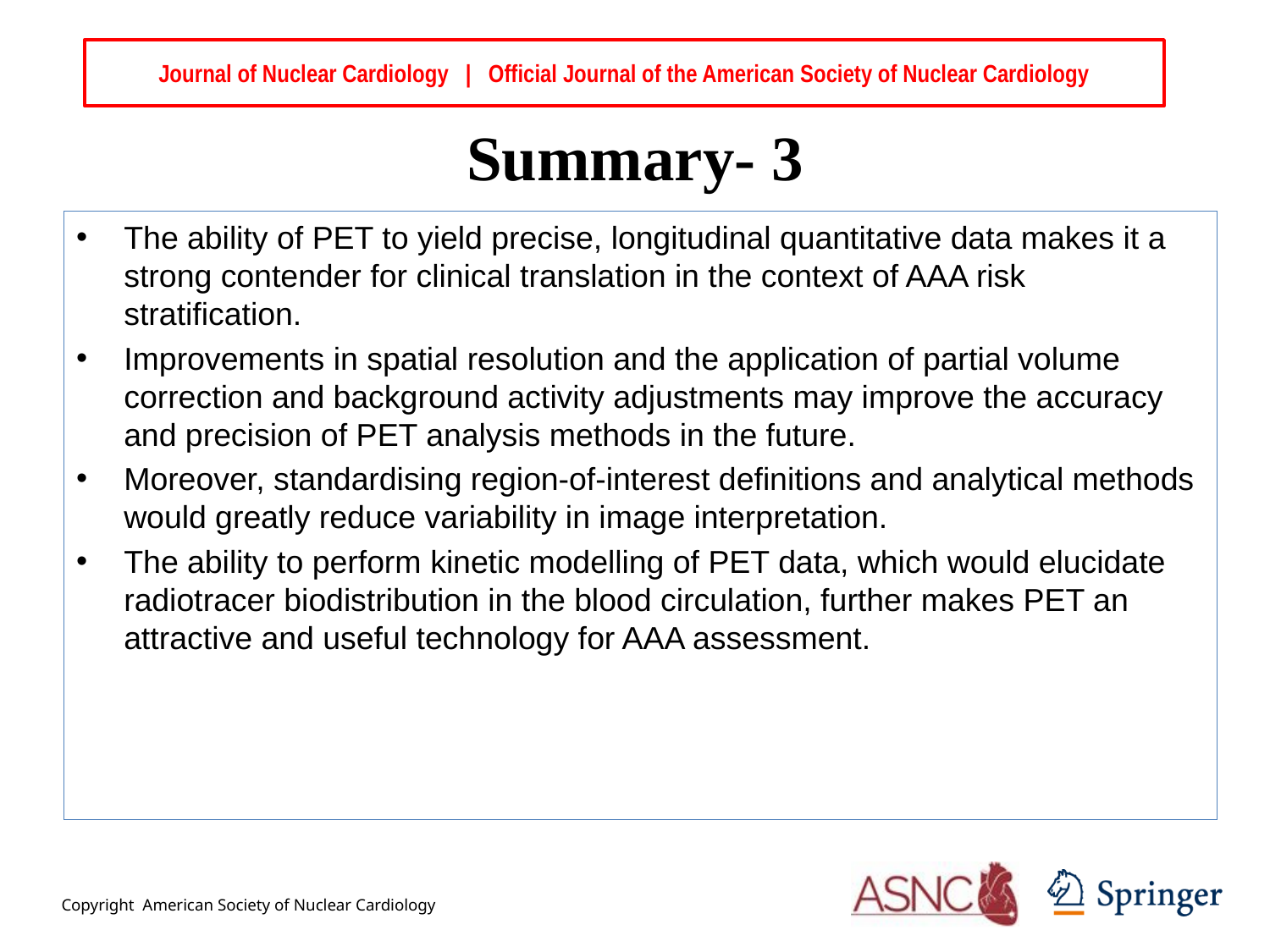

Journal of Nuclear Cardiology | Official Journal of the American Society of Nuclear Cardiology
# Summary- 3
The ability of PET to yield precise, longitudinal quantitative data makes it a strong contender for clinical translation in the context of AAA risk stratification.
Improvements in spatial resolution and the application of partial volume correction and background activity adjustments may improve the accuracy and precision of PET analysis methods in the future.
Moreover, standardising region-of-interest definitions and analytical methods would greatly reduce variability in image interpretation.
The ability to perform kinetic modelling of PET data, which would elucidate radiotracer biodistribution in the blood circulation, further makes PET an attractive and useful technology for AAA assessment.
Copyright American Society of Nuclear Cardiology

## Slide 6
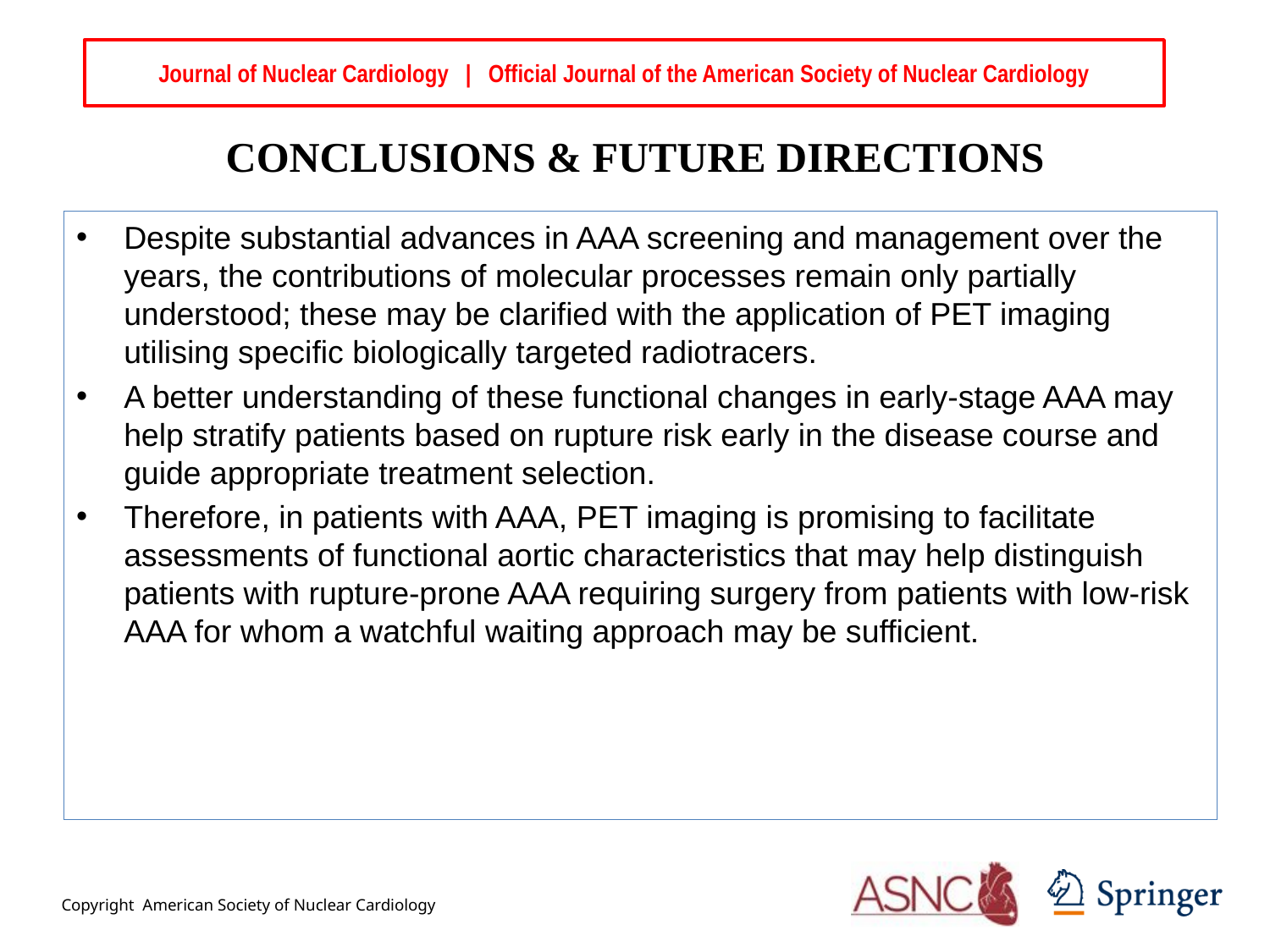

Journal of Nuclear Cardiology | Official Journal of the American Society of Nuclear Cardiology
# CONCLUSIONS & FUTURE DIRECTIONS
Despite substantial advances in AAA screening and management over the years, the contributions of molecular processes remain only partially understood; these may be clarified with the application of PET imaging utilising specific biologically targeted radiotracers.
A better understanding of these functional changes in early-stage AAA may help stratify patients based on rupture risk early in the disease course and guide appropriate treatment selection.
Therefore, in patients with AAA, PET imaging is promising to facilitate assessments of functional aortic characteristics that may help distinguish patients with rupture-prone AAA requiring surgery from patients with low-risk AAA for whom a watchful waiting approach may be sufficient.
Copyright American Society of Nuclear Cardiology
